# pyPaSWAS: Python-based multi-core CPU and GPU sequence alignment

**DOI:** 10.1371/journal.pone.0190279

**Published:** 2018-01-02

**Authors:** Sven Warris, N. Roshan N. Timal, Marcel Kempenaar, Arne M. Poortinga, Henri van de Geest, Ana L. Varbanescu, Jan-Peter Nap

**Affiliations:** 1 Expertise Centre ALIFE, Institute for Life Science & Technology, Hanze University of Applied Sciences Groningen, Groningen, the Netherlands; 2 Applied Bioinformatics, Wageningen University and Research, Wageningen, the Netherlands; 3 Parallel and Distributed Systems, Delft University of Technology, Delft, the Netherlands; UMR-S1134, INSERM, Université Paris Diderot, INTS, FRANCE

## Abstract

**Background:**

Our previously published CUDA-only application PaSWAS for Smith-Waterman (SW) sequence alignment of any type of sequence on NVIDIA-based GPUs is platform-specific and therefore adopted less than could be. The OpenCL language is supported more widely and allows use on a variety of hardware platforms. Moreover, there is a need to promote the adoption of parallel computing in bioinformatics by making its use and extension more simple through more and better application of high-level languages commonly used in bioinformatics, such as Python.

**Results:**

The novel application pyPaSWAS presents the parallel SW sequence alignment code fully packed in Python. It is a generic SW implementation running on several hardware platforms with multi-core systems and/or GPUs that provides accurate sequence alignments that also can be inspected for alignment details. Additionally, pyPaSWAS support the affine gap penalty. Python libraries are used for automated system configuration, I/O and logging. This way, the Python environment will stimulate further extension and use of pyPaSWAS.

**Conclusions:**

pyPaSWAS presents an easy Python-based environment for accurate and retrievable parallel SW sequence alignments on GPUs and multi-core systems. The strategy of integrating Python with high-performance parallel compute languages to create a developer- and user-friendly environment should be considered for other computationally intensive bioinformatics algorithms.

## Background

A major challenge in applied bioinformatics is the adoption of advanced high-performance tools and algorithms by end-users with possibly low-to-moderate software engineering skills in the context of their biological research questions. Earlier, we presented the CUDA-only application PaSWAS [1] that performs Smith-Waterman (SW) sequence alignment for any type of sequence on NVIDIA-based GPUs. PaSWAS is relatively fast and combined the accuracy of SW alignment with the possibility to retrieve alignment information relevant for biologists, in contrast to most other parallel SW implementations. Yet, adoption of PaSWAS can be improved: it may be too complex to install and use. In addition, use of the application was limited to NVIDIA-based hardware. Also in other cases, the adoption of highly promising tools and approaches is slower than expected. For example, the *de novo* assembly tool CloudBrush [2] uses MapReduce on Hadoop [3,4], but has seen no biological applications yet. The three versions of the NVIDIA CUDA-based sequence alignment tool CUDASW++ [5–7] are cited often, but citations deal in the larger majority with novel software implementations. The latest version CUDASW++ 3 [7], for example, has been cited 116 times (as of July 2017) but none of these citations deal with a direct biological question. The lack of adoption of promising new developments in algorithms and hardware may indicate that we as developers underestimated the complexity of setting up and running such a new application, especially when it is limited to a certain platform.

Another important limiting factor in the use of PaSWAS is the absence of the affine gap penalty. This scoring method produces biologically more relevant alignments than using only a gap open penalty [8]. It is therefore an important feature missing from the Smith-Waterman implementation in PaSWAS.

To improve the accessibility and use of PaSWAS, we have developed an entirely new software package, pyPaSWAS, based on OpenCL and CUDA integrated with Python. Python is a platform-independent programming language, with many libraries appropriate for bioinformatics, such as BioPython [9] and SciPy [10]. The open compute language OpenCL [11] is the current standard for clusters and/or multi-core CPU/GPU’s to speed-up analyses up to several orders of magnitude compared to single core CPU versions. OpenCL is similar to CUDA, but is supported by a growing number of manufacturers, including Intel, NVIDIA, Apple and IBM. By supporting both CUDA and OpenCL, pyPaSWAS runs on many platforms, including CPUs, GPUs other than NVIDIA-based GPUs and so-called accelerator cards. We integrated the PaSWAS CUDA [1] and OpenCL codebases with Python through pyCUDA [12] and pyOpenCL [12]. The original PaSWAS code was extended to add support for the affine gap penalty scoring method [8]. The result is a versatile Python-based user-friendly application for SW sequence alignment on a variety of multi-core systems. We propose this strategy as showcase for the integration of new software based on these compute languages with common programming tools such as Python to promote the adoption of advanced tools and applications in applied bioinformatics.

## Implementation

The new software package pyPaSWAS is implemented in Python (2.7 and up) and is run from the command line. It uses the libraries pyOpenCL [12] and pyCUDA [12] for device handling, memory allocation and kernel invocations to run the core PaSWAS Smith-Waterman code on the parallel device. pyPaSWAS depends on OpenCL 1.2+ [11] or Cuda 2.0+ [13], numpy[14] and biopython [9]. All other processing, such as Input / Output handling, logging and exception handling, are done in standard Python. The SeqIO class from bioPython [9] is used for file input. Its reference manual [15] lists all formats supported, including multi-fasta, genbank and fastq. Input file formats not supported by bioPython can be implemented by extending the Core.Reader class. Output can be formatted in a custom format by extending the Core.DefaultFormatter class. The Core.SAMFormatter class generates SAM output and can also be used as template for other custom output. The SAM descriptors (Table 1) are particularly useful for further processing output data. File-based configurations allow for storing settings and consistent reruns of the application. The user can supply appropriate scoring values for alignment, for example substitution matrices, to adjust the analyses to the desired specifications. The Core.Score module can be adjusted to support any 255 by 255 scoring matrix. The accompanying wiki [16] provides a complete description of the command line arguments as well as examples of how to run pyPaSWAS.

**Table 1:** Options in PyPaSWAS for selecting and filtering the alignments.

Filter name*	Value range**	Default	SAM descriptor	Description
lower_limit_score	0.0 < x < = 1.0	1.0		Allows for more hits per alignment. All hits with a score within this fraction of the maximum score found are reported. Used during the backtracing procedure for reducing the number of alignments to be processed.
minimum_score	0 < x	30	AS:i:	Minimum score of an alignment. Used during the backtracing procedure for reducing the number alignments to be processed..
filter_factor	0.0 < x < = 1.0	0.2	AS:i:	For each alignment the theoretical maximum score is calculated: length of the shortest sequence times the maximum score for a match (eg. the score for a perfect alignment). Only alignments with a score above filter_factor times this theoretical maximum score are returned.
query_coverage	0.0 < = x < = 1.0	0.2	QC:f:	Minimum fraction of the query covered in the alignment
query_identity	0.0 < = x < = 1.0	0.2	QI:f:	Minimum fraction of matches relative to the query
relative_score	0.0 < x < = score match	2.0	RS:f:	Minimum score relative to the shortest sequence. A full match will give a relative score of the match score, for DNA/RNA sequences the default is 5.0
base_score	0.0 < x < = score match	2.0	BS:f:	Score of the alignment divided by the length of the alignment.

*Filter name: all parameters available for filtering;

** value range: the boundaries for the settings of the corresponding parameter.

The structure of CPU hardware differs from GPU hardware and running OpenCL code designed for GPUs is not optimal [17]. Therefore, two OpenCL versions based on the CUDA-based implementation in PaSWAS were developed, one for GPUs and one for CPUs [18]. The latter makes better use of CPU hardware for faster sequence alignments. The two OpenCL implementations differ from the previous CUDA implementation only in the use of specific OpenCL calls; no changes have been made to the underlying algorithms.

The OpenCL implementation runs on multi-core hardware supporting OpenCL 1.2, such as Intel/AMD CPUs and accelerator cards (GPUs and Xeon Phi). With the CUDA implementation, pyPaSWAS runs on all NVIDIA GPUs with compute capability 1.2 and above, which includes support for all recent NVIDIA GPUs, including laptop versions, Teslas and the GTX-based cores. By default pyPaSWAS runs on the CPU using the CPU-optimized OpenCL code. To use other parallel devices than the CPU, the user changes the configuration or selects the appropriate device through command line options.

pyPaSWAS opens the platform selected, sets the appropriate memory usage and other parameters relevant for the parallel device automatically, based on settings and data to be analyzed. pyPaSWAS allows for fine grained control over the use of the parallel device, such as memory usage and number of compute cores to be used. CPU hardware allows for limiting the number of cores used by an application. This enables using the computer for other tasks and is necessary when pyPaSWAS runs in a cluster environment. This fine-grained control level presents a major improvement over the earlier PaSWAS [1] in addition to the integration with Python. All options are listed on the wiki-page [16] and are accessible through the command line (‘-h’).

As its predecessor [1], pyPaSWAS documents all alignment details and allows for filtering of the resulting alignments. Parameters for filtering are listed in Table 1. Parameters can be set through the configuration file or through command line options. This gives the ability to select which hits are relevant and will be sent to the output file. The scoring value and all related values, such as query coverage, are present in the output and can also be used to filter the results further afterwards (Table 1).

### Affine gap penalty

For biologically more relevant alignments, the affine gap penalty method [8] scores the opening of a gap differently than for extending a gap. The original PaSWAS code only supported the gap penalty scoring method, which means that each gap has the same score, no matter how many gaps are in front of it. The affine gap penalty implementation requires a scoring matrix M, to keep track of the match scores and scoring matrices *I* and *J* to keep track of the scores for gaps in the target (*I*) and query (*J*) sequences. The PaSWAS implementation of the direction matrix has been extended to record which of the three matrices resulted in the highest score. The downside of using an affine gap method is that it requires creating two additional matrices (*I*, *J*) of the same size as the already existing scorings matrix (*M*). This means that a 100x100 sequence alignment using the affine gap requires not 10,000 scoring values, but 30,000 scorings values. Next to an increase in memory usage, additional calculations compared to the original SW implementation are needed, making the affine gap method slower (see S3 Report). The affine gap penalty method is not required in all cases, for example when the gaps originated from technical (NGS) issues and do not have any biological meaning. In such cases, the PaSWAS code is used to perform a SW-alignment without a gap extension penalty. The user controls the use of the affine gap penalties by setting a value other than zero for the gap extension penalty (the ‘-g’ option).

## Results and discussion

The performance of pyPaSWAS is expressed as the time required for the number of SW alignments processed. Six different configurations were tested for performance (Table 2), with variations in hardware (Intel or NVIDIA), parallel device (CPU or GPU), code usage (optimized for CPU or GPU), number of cores used and the language involved (OpenCL or CUDA).

**Table 2:** Configurations for testing the performance of pyPaSWAS.

Configu-ration	Hardware	Parallel device	Code optimized for	Nr. of cores	Language	Time for 2720 alignments (s)	GCUPS**	Speedup compared to F
**A**	Intel i7	CPU	CPU	1	OpenCL	119.2	0.70	0.21
**B**				8		106.4	0.82	0.21
**C**			GPU	1		812.6	0.10	0.03
**D**				8		192.3	0.44	0.12
**E**	NVIDIA GTX 1070	GPU	GPU	1920	OpenCL	57.8	1.48	0.36
F*					CUDA	17.6	4.64	1.00

*Configuration (F) is equivalent to the earlier PaSWAS [1], and is therefore used as reference here. The last two columns give the amount of time spent on the largest set of alignments in the performance analysis and the speedup compared to the configuration (F).

**GCUPS: giga cell updates per second.

In all cases, pyPaSWAS was run on a standard desktop (Intel i7 -2600K) running Ubuntu 16.02 and holding an NVIDIA GeForce GTX 1070 GPU. Timing of alignments was done by determining the run time of the application between first and last API calls to the Python libraries (either pyOpenCL or pyCUDA), so overhead such as file I/O is not taken into account. The full report is in S1 Report. Performance analysis with the same data set on a standard laptop is in S2 Report.

**Fig 1:**
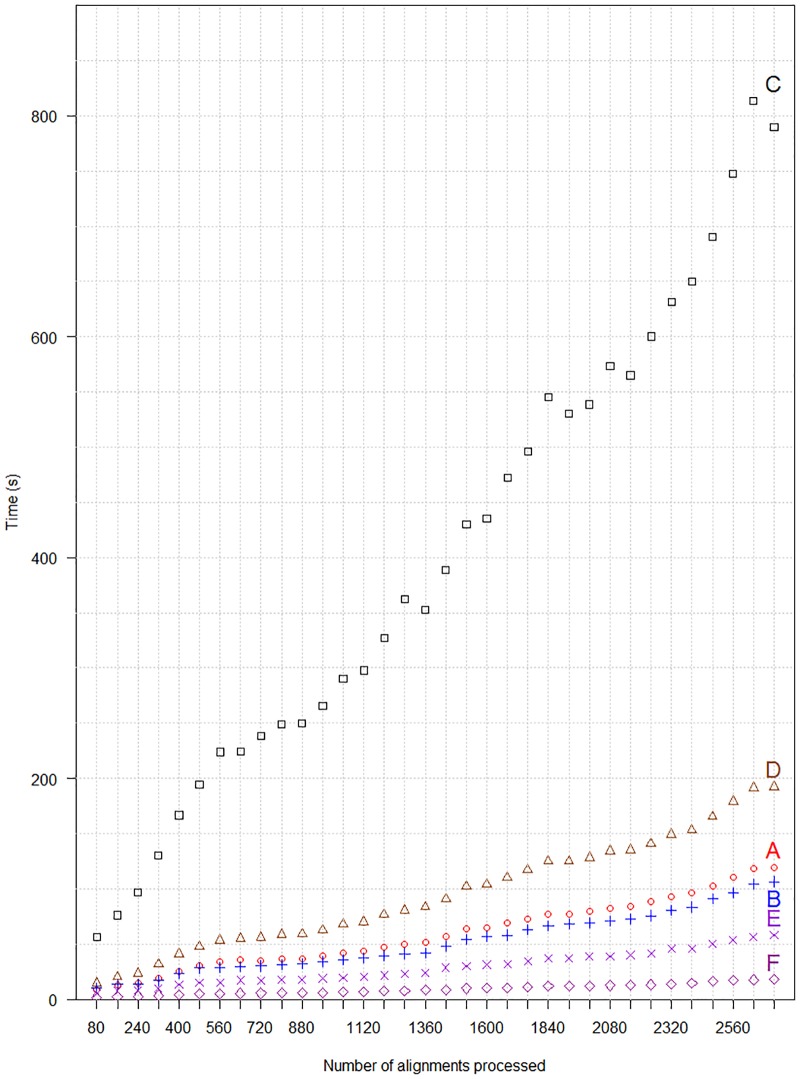
Performance of six different configurations for pyPaSWAS in Smith Waterman (SW) alignments. The time required (Y-axis) for processing an incremental number of alignments (X-axis) is plotted. For details of the different configurations A-F see Table 2.

As test set for the performance analysis of pyPaSWAS on the different hardware configurations, the Ankyrin repeat protein set from the domestic dog (*Canis lupus familiaris*; CanFAM 3.1, GCA_000002285.2), consisting of 348 proteins was used. For the performance analysis, the eight proteins not labeled ‘PRED’ were selected and aligned to an increasing number of proteins from the total data set. The time required to calculate the increasing number of SW alignments by the six configurations is shown in Fig 1. The time for performing the maximum of 2720 sequence alignments is also given in Table 2. As these protein sequences differ in length, it is common to indicate the speed of the SW computations in giga cell updates per second (GCUPS) to create an performance indicator independent of sequence length. The alignment output itself and the biological context were not considered. In this example data set the CUDA implementation running on the GPU (F) is the fastest configuration and is 2.8 times faster than the OpenCL version optimized for the GPU (E). The data also shows that the fastest configuration (F) is 33.3 times faster than the for GPU optimized OpenCL version on single CPU core, showing the advantages of parallel processing of SW alignments on a GPU. The for CPUs optimized OpenCL version (B) is 1.8 times faster than the for GPUs optimized version (D) on the same CPU. This shows that creating an OpenCL version of an application optimized for a particular hardware platform can speed up the application further.

The performance tests using only a single core demonstrate the ability of pyPaSWAS to scale-down the number of cores used for the sequence alignments.

The CUDA version (configuration F) is faster than the OpenCL version on a GPU (configuration E), showing the added value of having a CUDA version in this case. There are several other reasons for having CUDA support in pyPaSWAS. In general, CUDA is faster than OpenCL [17]. Also, on some systems we tested, notably Apple Macs, OpenCL is not fully supported on NVIDIA GPUs, so CUDA is the only option available. Furthermore, several NVIDIA GPU products support only 32 bits memory allocation for OpenCL, which limits the amount of usable memory to 2 GB, but allow 64 bits memory for CUDA.

Analyses of the impact of the affine gap penalty on overall performance when the using the same data sets show that, on a desktop PC, all configurations are slower: from 1.14 times to 2.0 times slower (S3 Report). Combined with the fact that memory requirement is also three times higher, it is therefore opportune to make sure that the affine gap is relevant for the task at hand.

A major advantage of PaSWAS for biological analyses is that it documents all alignment details necessary for further analysis, in marked contrast to other parallel SW implementations that focus on computational speed of the best alignment[1]. When for example compared to CUDASW++ version 3.0 getting the alignment profile comes with a performance penalty of about 25x (119.0 GCUPS [7] compared to 4.64 GCUPS) on similar hardware. The novel implementation pyPaSWAS here presented is more versatile for biological analysis then the original PaSWAS code-base: not only full alignment details are stored and available for inspection, it also allows for gap extension penalties in scoring the alignment. In addition, the output can now also be formatted as a SAM file. Also, pyPaSWAS has more command line options and the output contains more relevant information, such as query coverage and query identity scores. The Python codebase enables bioinformatics researchers to add other output formats, store the alignments directly in a database or connect the application with workflow systems such as Galaxy [19]. In addition, the source repository holds configuration files to build Docker containers, including one Docker container with CUDA and OpenCL support, to allow for easy installation of pyPaSWAS and the required drivers and libraries.

As data volumes continue to grow and analyses tend to become more complex in every branch of bioinformatics, the added value of advanced high-performance IT solutions such as multicore CPUs and GPUs is transforming into a need for such solutions. Multicore CPUs for Blast [20] and BWA [21], cluster computing for Interproscan [22] and cloud infrastructure for a wide range of biomedical / bioinformatics applications are available [23]. High performance technology used in mathematics [24,25] and audio/video processing [26] rely on GPUs and OpenCL. Wider acceptance of OpenCL -based GPU applications in bioinformatics is likely to be promoted by packaging the C++ code for parallelization in a much more common used language such as Python as demonstrated here. The pyPaSWAS integration of Python with OpenCL should promote further use of advanced algorithms in bioinformatics. Given this successful showcase for the integration of OpenCL with new or existing software in Python, it could be considered to port bioinformatics algorithms that make use of advanced high performance technology to Python, R [27], Matlab [28] or Java [29] in a way similar to pyPaSWAS. This will promote use, maintenance and development of high performance implementations of bioinformatics applications further. Such an approach could benefit for example algorithms for genome wide association studies [30], eQTL analyses [31] or phylogenetics [32].

## Conclusion

pyPaSWAS is the implementation in Python of a general-purpose Smith-Waterman alignment supporting both the basic gap penalty method as well as the affine gap penalty method. The application runs fast on many multi-core systems, including GPUs and Xeon Phis, while still offering the desired flexibility to inspect any given alignment and all its parameters. The Python-based application will increase the use and utility of the parallel SW approach of PaSWAS. The smooth integration of Python with the much more complex languages OpenCL and CUDA for parallel execution of the SW algorithm makes pyPaSWAS easier to develop and maintain than its predecessor. The relative ease of Python, as well as the much larger community of programmers in Python, is likely to promote adoption and use, as well as facilitate addition of novel features to pyPaSWAS.

## Supporting information

S1 Report pyOpenCL and pyCUDA performance data (Desktop system). Full report on the timing measurement of the protein alignment analyses, run on a standard desktop PC.
(PDF)

S2 Report pyOpenCL and pyCUDA performance data (Laptop). Full report on the timing measurement of the protein alignment analyses, run on a high-performance laptop.
(PDF)

S3 Report Analyses of the impact of the affine gap penalty on overall performance (Desktop system). (PDF)
