# Supplementary material for: pyPaSWAS: Python-based multi-core CPU and GPU sequence alignment
Source: PLoS One. 2018 Jan 2;13(1):e0190279. doi: 10.1371/journal.pone.0190279 (PMC5749749; doi:10.1371/journal.pone.0190279)
Supplement: S2 Report — Full report on the timing measurement of the protein alignment analyses, run on a high-performance laptop. (PDF) [file pone.0190279.s002.pdf]

pyOpenCL and pyCUDA performance data

Laptop

These configurations are used:

A) CPU, cpu-OpenCL, 1 core
B) CPU, cpu-OpenCL, 8 cores
C) CPU, gpu-OpenCL, 1 core
D) CPU, gpu-OpenCL, 8 cores
E) GPU, gpu-OpenCL
F) GPU, CUDA

Definitions:

- GPU: NVIDIA GT 650M
- CPU: Intel i7
- CUDA: PaSWAS Smith-Waterman code base
- gpu-OpenCL: for GPU optimized OpenCL code base
- cpu-OpenCL: for CPU optimized OpenCL code base

Timing measurements

We would like to show the ability of pyPaSWAS to align protein sequences on different devices using one of the three possible implementions and also perform performance (timing) measurements on these alignments. There is no underlying biological question. Protein sequences were chosen and not DNA/RNA, because the latter are also in the PaSWAS paper.

The timing is done on 8x10, 8x20, 8x30, . . . , 8x340 protein alignments.

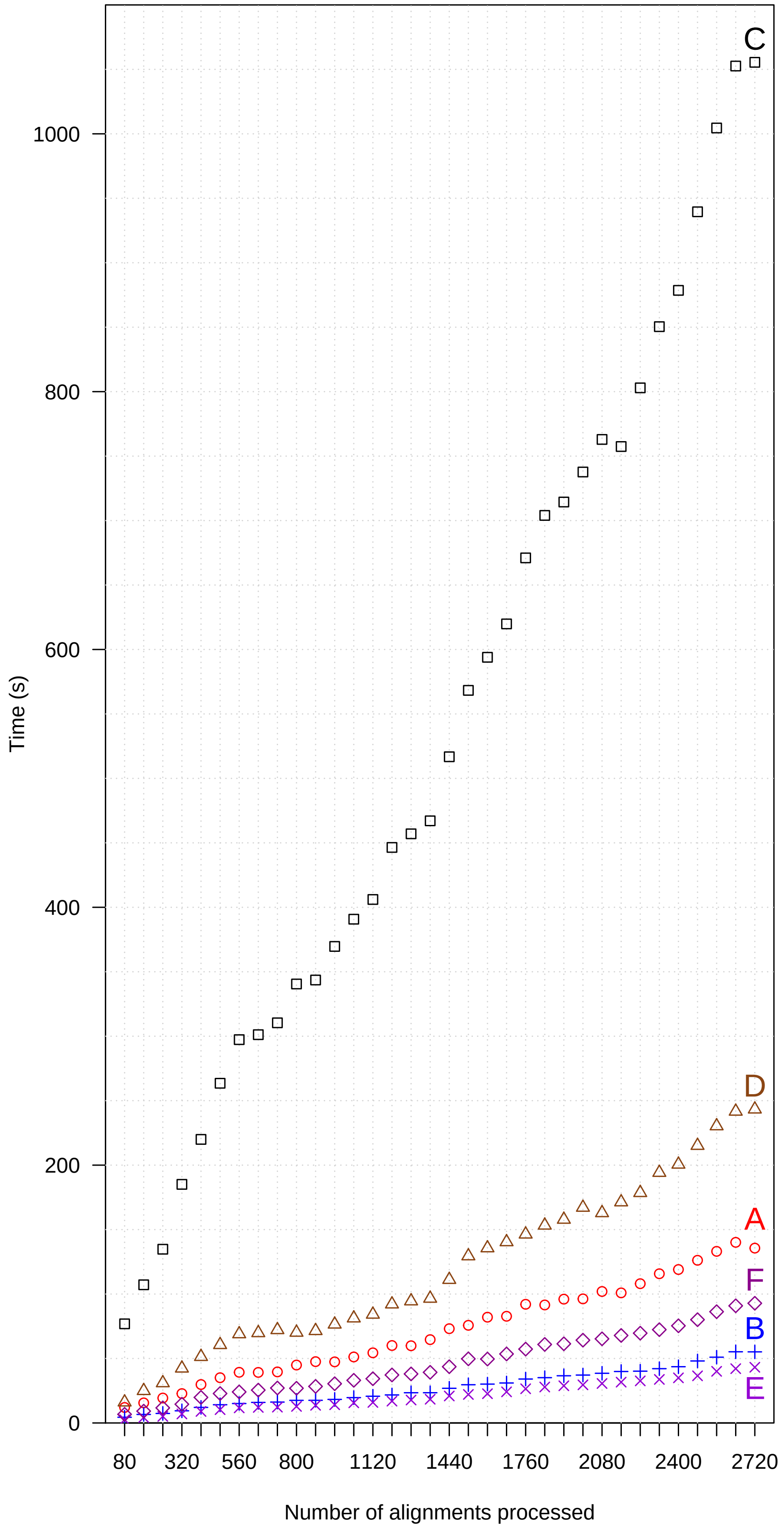

Time (s)
1000
800
600
400
200
0
80
320
560
800
1120
1440
1760
2080
2400
2720
Number of alignments processed
C
D
A
F
B
E

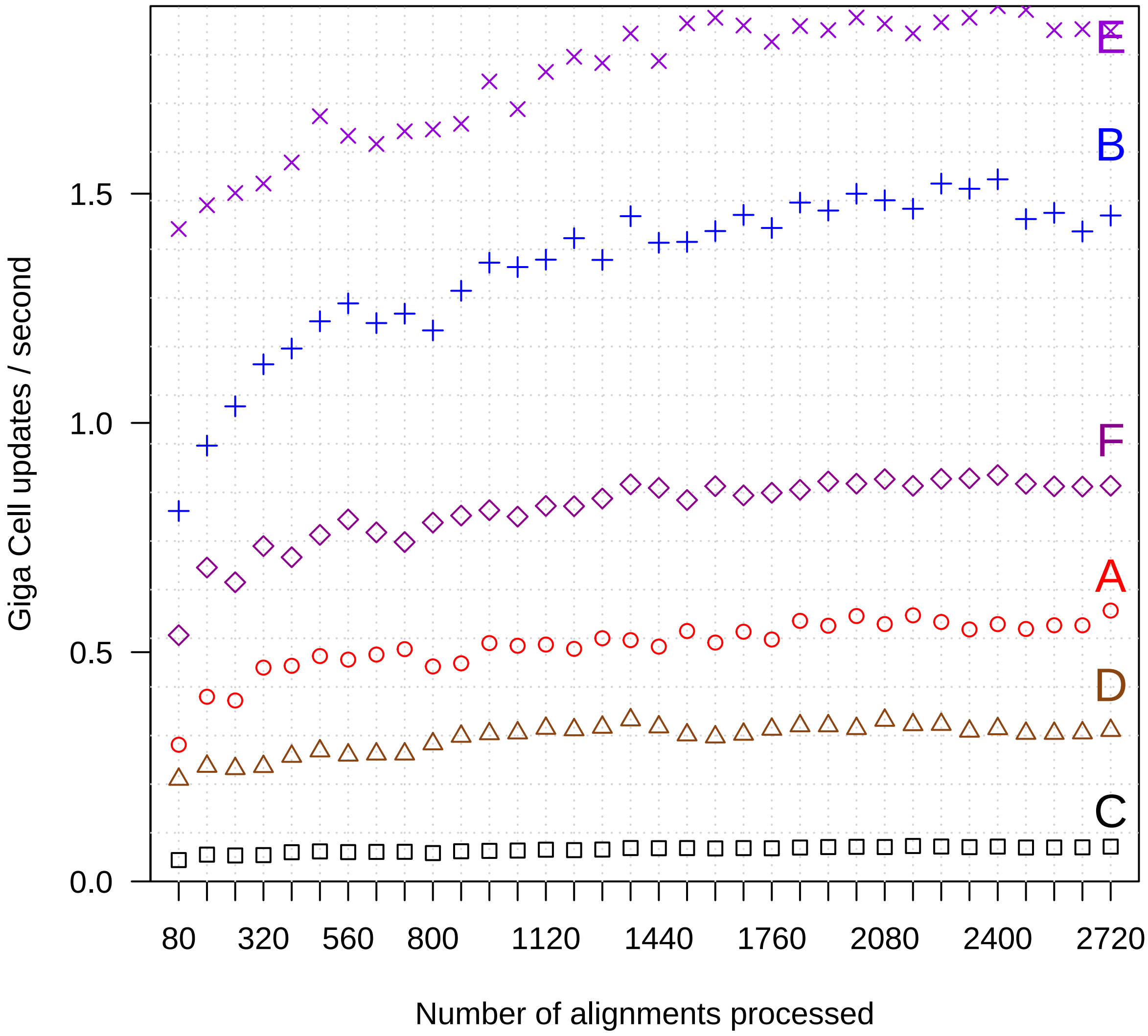

Comments:

- The lines are not completely straight because:
  1) lengths of sequences vary,
  2) CUDA / OpenCL devices perform optimalizations on code and execution
  3) fluctations due to other processes running on the devive
- The axes and data are chosen in such a way that using the CPU for Smith-Waterman on a single core is the (much) worse performaning setup (= implementation + device + data) of all. It also shows that parallel processing on the GPU in these setups is the fastest way of doing SW.

Speed-up compared to CUDA

To show the speed-up of each of the configuration compared to the GPU, CUDA configuration, each timing measurement is compared to the timing of the GPU, CUDA configuration.

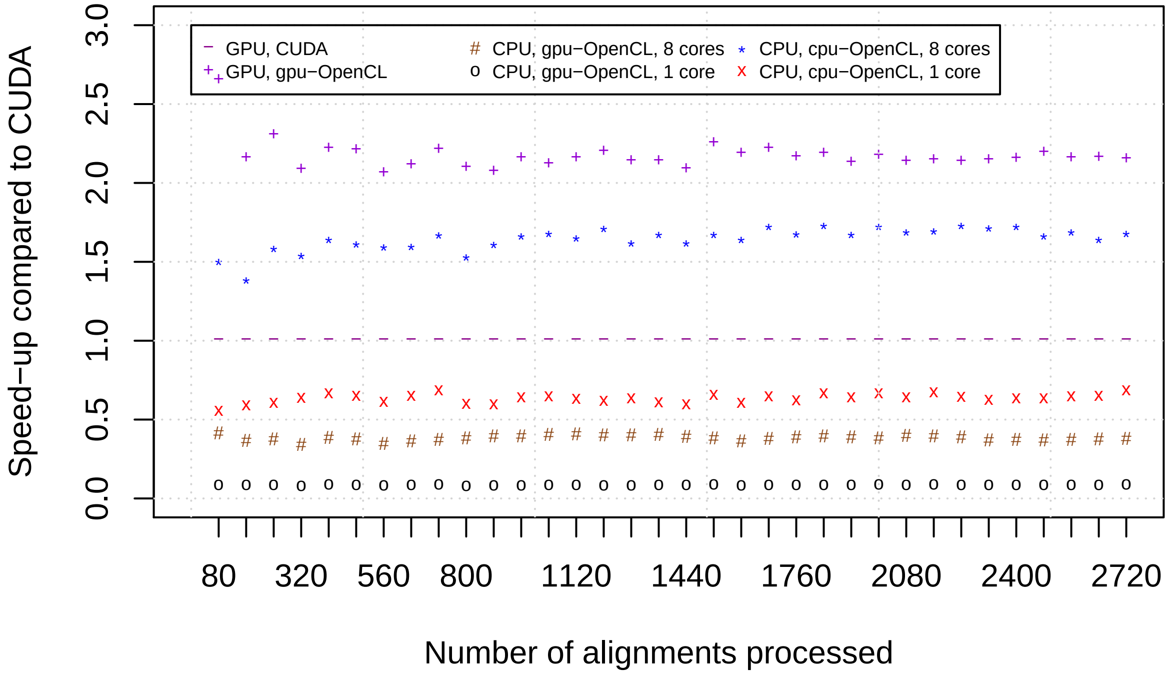

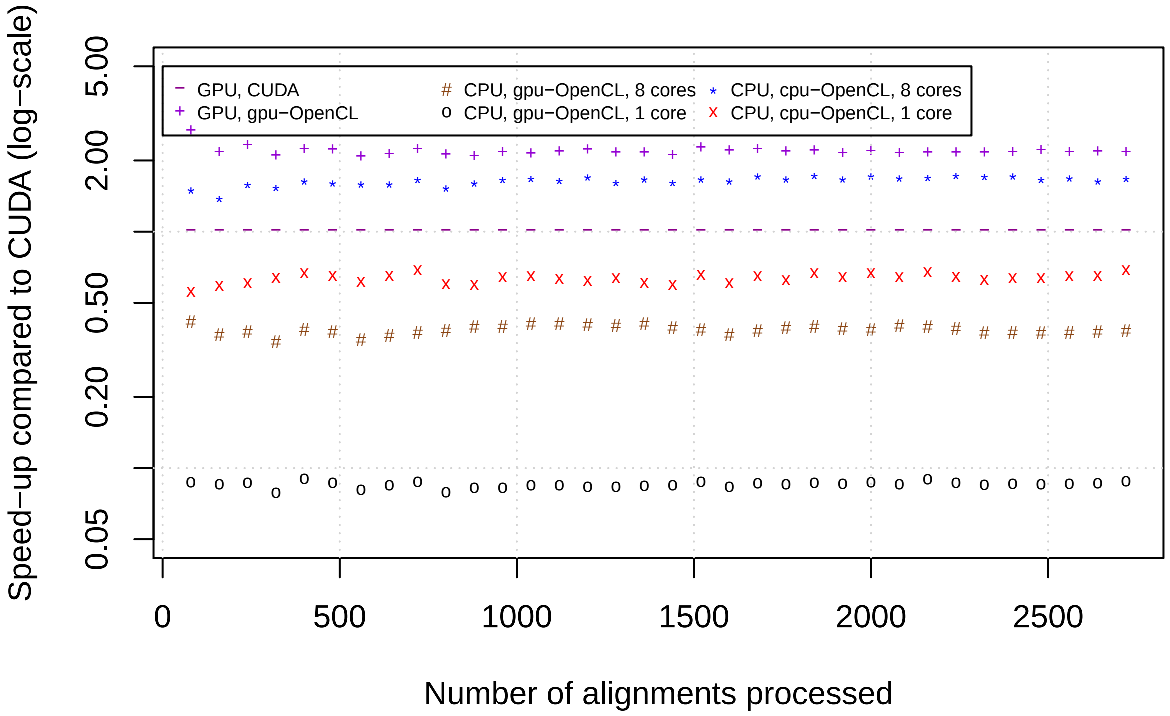

Comments:

- GPU + OpenCL is the fasted in this setup
- plot quantifies speed differences between setup with CUDA and other setups
- Speed-up for each of the setups is stable across different number of alignments performed: this plot can be summarized in a table:

Configuration	Timing	GCUPS	Speedup
GPU, CUDA	92.84	0.89	1.00
GPU, gpu-OpenCL	43.21	1.91	2.65

Configuration	Timing	GCUPS	Speedup
CPU, gpu-OpenCL, 8 cores	243.35	0.35	0.41
CPU, gpu-OpenCL, 1 core	1055.47	0.08	0.09
CPU, cpu-OpenCL, 8 cores	55.21	1.53	1.73
CPU, cpu-OpenCL, 1 core	140.11	0.59	0.68
