# Supplementary material for: pyPaSWAS: Python-based multi-core CPU and GPU sequence alignment
Source: PLoS One. 2018 Jan 2;13(1):e0190279. doi: 10.1371/journal.pone.0190279 (PMC5749749; doi:10.1371/journal.pone.0190279)
Supplement: S3 Report — (PDF) [file pone.0190279.s003.pdf]

pyOpenCL and pyCUDA performance data for gap extension

Desktop system

These configurations are used:

A) CPU, cpu-OpenCL, 1 core
B) CPU, cpu-OpenCL, 8 cores
C) CPU, gpu-OpenCL, 1 core
D) CPU, gpu-OpenCL, 8 cores
E) GPU, gpu-OpenCL
F) GPU, CUDA

Definitions:

- GPU: GeForce GTX 650
- CPU: Intel(R) Core(TM) i7-3770 CPU @ 3.40GHz
- CUDA: PaSWAS Smith-Waterman code base
- gpu-OpenCL: for GPU optimized OpenCL code base
- cpu-OpenCL: for CPU optimized OpenCL code base

System:

- Ubuntu 15.04
- Desktop PC

Performance penalty for gap extension

To show the performance penalty of using the gap extension penalty, each SW run is compared to the timing of doing a SW with a gap extension penalty. Memory usage, data sets and other setings were kept the same to make sure only the performance penalty of the introduction of the gap extension penalty was measured.

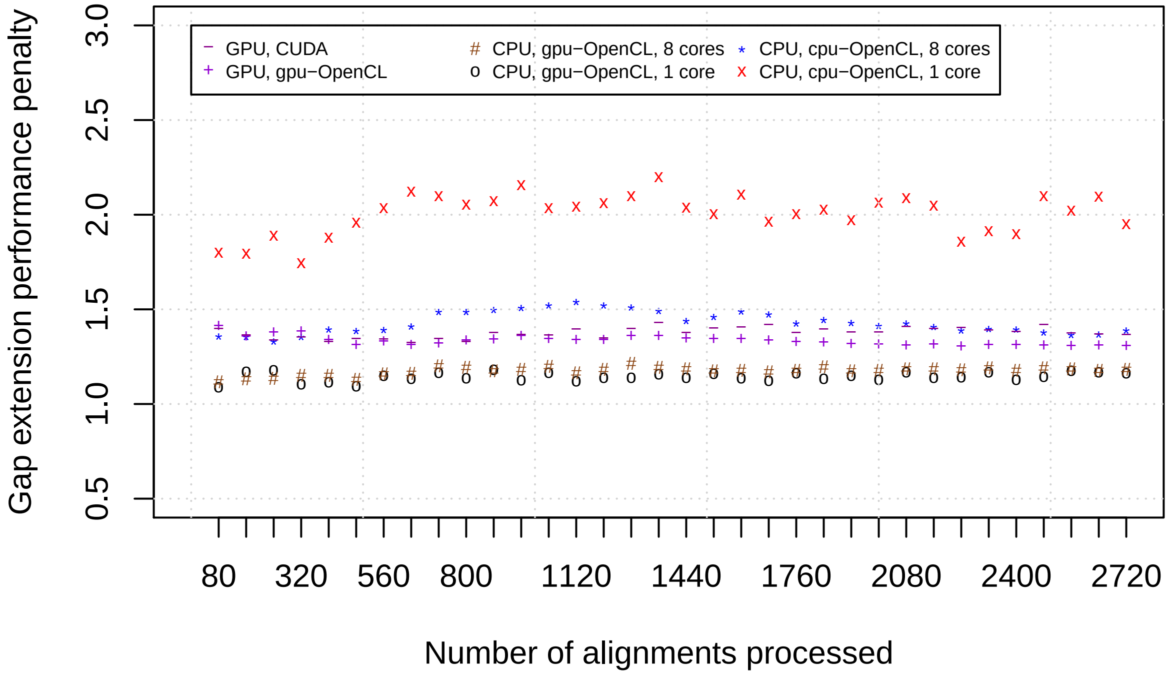

Average slow down when using gap extension penalty

Configuration	Average slow down
GPU, CUDA	1.3688687
GPU, gpu-OpenCL	1.3284135
CPU, gpu-OpenCL, 8 cores	1.1748376
CPU, gpu-OpenCL, 1 core	1.1427274
CPU, cpu-OpenCL, 8 cores	1.4348
CPU, cpu-OpenCL, 1 core	2.0043717
